# Controlling Structure and Dimensions of a Disordered Protein via Mutations

**DOI:** 10.1021/acs.biochem.9b00678

**Published:** 2019-09-26

**Authors:** Sneha Munshi, Divya Rajendran, Samyuktha Ramesh, Sandhyaa Subramanian, Kabita Bhattacharjee, Meagha Ramana Kumar, Athi N. Naganathan

**Affiliations:** Department of Biotechnology, Bhupat & Jyoti Mehta School of Biosciences, Indian Institute of Technology Madras, Chennai 600036, India

## Abstract

The dimensions of intrinsically disordered proteins (IDPs) are sensitive to small energetic-entropic differences between intramolecular and protein–solvent interactions. This is commonly observed on modulating solvent composition and temperature. However, the inherently heterogeneous conformational landscape of IDPs is also expected to be influenced by mutations that can (de)stabilize pockets of local and even global structure, native and non-native, and hence the average dimensions. Here, we show experimental evidence for the remarkably tunable landscape of IDPs by employing the DNA-binding domain of CytR, a high-sequence-complexity IDP, as a model system. CytR exhibits a range of structure and compactness upon introducing specific mutations that modulate microscopic terms, including main-chain entropy, hydrophobicity, and electrostatics. The degree of secondary structure, as monitored by far-UV circular dichroism (CD), is strongly correlated to average ensemble dimensions for 14 different mutants of CytR and is consistent with the Uversky–Fink relation. Our experiments highlight how average ensemble dimensions can be controlled via mutations even in the disordered regime, the prevalence of non-native interactions and provide testable controls for molecular simulations.

Mutational perturbation of protein structures reveals position-specific and context-dependent information on protein structure network, folding, function, allostery, and epistasis.^1–4^ A majority of studies have been performed on ordered proteins with a compact hydrophobic core and a well-defined three-dimensional structure.^5–8^ However, elementary considerations of the intraprotein interaction or contact network highlight that mutations should not only modulate the folded versus unfolded state equilibrium but also tune the relative population of intermediate and excited states and thus the overall dimensions of the native ensemble.^4,9^ Even small volume fluctuations are expected as the packing interaction can be weakened via specific mutations without changing the overall structure.^12^ Testing this on folded proteins is a huge challenge as small changes in dimensions are difficult to extract from conventional experiments.

Intrinsically disordered proteins (IDPs), on the other hand, provide a wonderful testing bed for probing mutation-driven structural perturbations.^13^ High-sequence-complexity IDPs, i.e., those protein sequences that are disordered despite exhibiting little compositional bias, are more interesting as they are expected to populate pockets of local structure, rich in native or non-native interactions (deduced from the folded conformation), apart from fully disordered states. For example, this can be observed in studies of denatured states of folded proteins.^14–16^ It should, therefore, be possible to tune the dimensions of natural IDPs through small perturbations of basic thermodynamic factors including backbone conformational entropy, hydrophobicity, and charge–charge interactions, that (de)stabilize pockets of structure.

The CytR DNA-binding domain (referred to as CytR hereon) is a high-sequence-complexity IDP that acquires a compact three-helix bundle structure in the presence of DNA (Figure 1A).^17^ The conformational ensemble of CytR is sensitive to temperature,^18^ salt,^19^ and DNA,^17,19^ all of which arise from a combination of destabilizing electrostatics (Figure 1B) and weak packing in the folded conformation.^20^ In this work, we control the dimensions of the disordered CytR via a combination of folded structure and sequence-alignment-based expectations, and rational engineering. Though we observe nonintuitive effects, a likely manifestation of non-native interactions within the disordered ensemble, we find a strong correlation between secondary structure and the apparent Stokes radius (*R*_s_), a first such observation in IDPs.

We have shown earlier that the P33A mutation in CytR reduces the secondary structure content by reducing the population of an excited folded-like conformation.^21^ Main-chain entropic considerations^22^ suggest that, if an amino acid with a higher flexibility is introduced at the same position, the secondary structure content is expected to be even lower, arising from the larger entropic destabilization of residual structure. True to this, the thermal dependence of the P33G mutant’s CD signal is flat (blue in Figure 2A) unlike the weak structural transitions observed in the WT or the P33A mutant (red and green in Figure 2A, respectively). In addition to the secondary structure content, the mean dimensions as measured by *R*_s_ (in a calibrated size-exclusion chromatography column^20^), shows a trend where P33G is more expanded than the WT (Figure 2B). Another interesting position is residue K35 located in the electrostatically frustrated binding site of the folded conformation with a number of positive charges around it (Figure 1B). Eliminating this positive charge through the K35Q mutant not only enhances the secondary structure content (though only marginally) but also reduces the dimensions of the ensemble (cyan in Figure 2).

Similarly, we have shown recently that the double mutant A29V/A48M (DM; that introduces two large hydrophobic substitutions in the protein core) promotes collapse of the disordered ensemble into a compact folded ensemble while simultaneously increasing the secondary-structure content (mutations identified via sequence comparisons; magenta in Figure 2 and Supporting Figure S1).^20^ Building on the folded-like conformation of the DM, we engineered two other mutants, Quad (A29V/A48M/R43A/K46A; black in Figure 2) and Pent (A29V/A48M/R43A/K46A/R28A; gray in Figure 2), that further eliminate the residual electrostatic frustration. These two mutants again promote structure and compaction while differentially affecting the secondary structure content. However, our attempts to rationally engineer structure or loss of structure were not always successful. For example, the A29V mutant enhances the structure-compactness only in the presence of the R28Q substitution and not otherwise (dark cyan in Figure 3A). Thus, collapse and structure acquisition in polymer chains is an emergent property arising from long-range many-body effects that can be engineered not only via hydrophobicity (as shown before for the DM) but also via a combination of hydrophobicity and electrostatics (A29V/R28Q mutant).

Interestingly, a number of single-point mutations (R28Q, A29V, D34S, R43N, R43E, K46A, and A48M) modulate ensemble dimensions despite being disordered (Figure 3A,B). The average dimensions of CytR can be mutationally varied from ~22 Å in the P33G mutant to ~16 Å in the Pent mutant (Figure 3B; Figure S2 for the elution profiles). If one excludes DM, Quad, and Pent that induce large structural changes, the maximal change in Stokes radius is ~3.5 Å, which is still significant as it corresponds to ~40% reduction in the volume of the protein chain compared to the fully unfolded P33G mutant.

Mutations that eliminate unfavorable interactions (R43N, R43E, K46A) as expected from the folded conformation still result in an expansion of the ensemble, suggestive of non-native interactions in the disordered state (Figure 3B). The only exceptions to this are the R28Q and K35Q mutations that promote slight compaction and the D34S mutation that promotes slight expansion, upon elimination of unfavorable and favorable interactions, respectively (Figure 3B). Despite these varied outcomes on mutations, we find that it is possible to collapse all such variations into a single plot of ensemble dimensions versus secondary-structure content (Figure 3C; *r* = 0.96 and *p* <10^−9^). In fact, the dependence of relative CD signals on the relative molecular volumes agrees reasonably well with the Uversky-Fink relation^23^ (Figure S3). We also observe a similar correlation between dimensions and secondary-structure content at different salt concentrations for the CytR WT combining SEC and analytical ultracentrifugation data (Figure S4). These observations are strong evidence that the magnitude of the far-UV CD signal at 222 nm (in absolute units) for a given protein can be an intimate indicator of the molecular dimension even in the disordered regime,^24^ particularly for helical protein domains, and if appropriately calibrated.

Is the correlation between Stokes radius and secondary structure a manifestation of (de)population of only folded-like conformations upon mutation? To answer this, we modeled the conformational behavior of CytR and its mutants via the semiquantitative Wako–Saitô–Muñoz–Eaton (WSME) statistical mechanical model that considers an ensemble of 2^47^ microstates (47 being the length of the folded region of CytR)^25,26^ with contributions from packing interactions, electrostatics, and implicit solvation.^27,28^ Since the model is Go-like,^29^ it captures the effect of only those mutations that display native-structure-derived expectations (Figure 2 and Table S1). The predicted unfolding curves are very similar to experimental observations, both in terms of the order of stability and structure (Figure 4A), validating the model energetics and assumptions. All of the generated free-energy profiles display multiple minima whose relative populations are modulated on mutation (Figure 4B,C). This is also consistent with both explicit and implicit-solvent simulations of WT CytR that highlight a flat conformational landscape with numerous competing sub-states.^18,30^ In fact, even the folded-like DM displays broad, weakly cooperative, and probe-dependent unfolding.^20^ Taken together, our observations suggest that the apparent Stokes radius represents an effective ensemble average of numerous partially structured states.

The solvent sensitivity of IDP dimensions arises from small imbalances between intrachain and chain–solvent interactions.^15,16,31–34^ In this work, we tune this relative balance by introducing mutations in a high-sequence-complexity IDP, and thus indirectly affecting the solvent quality (as seen by the polymer chain) without modulating solvent composition. Our results establish that the average dimensions of CytR can be modulated via mutations in a continuous manner through both rational engineering and non-native effects. CytR and its mutants can thus serve as excellent model systems for simulations, and to particularly validate force-fields and water models without resorting to extreme solvent conditions or temperature.

## Supplementary Material

The Supporting Information is available free of charge on the ACS Publications website at DOI: 10.1021/acs.bio-chem.9b00678.

WSME model parameters, dimensions of CytR WT and DM from DLS measurements, size-exclusion chromatography elution profiles, the Uversky-Fink plot, and structural changes on ionic strength modulations (PDF)

Supporting Information

## Figures and Tables

**Figure 1:**
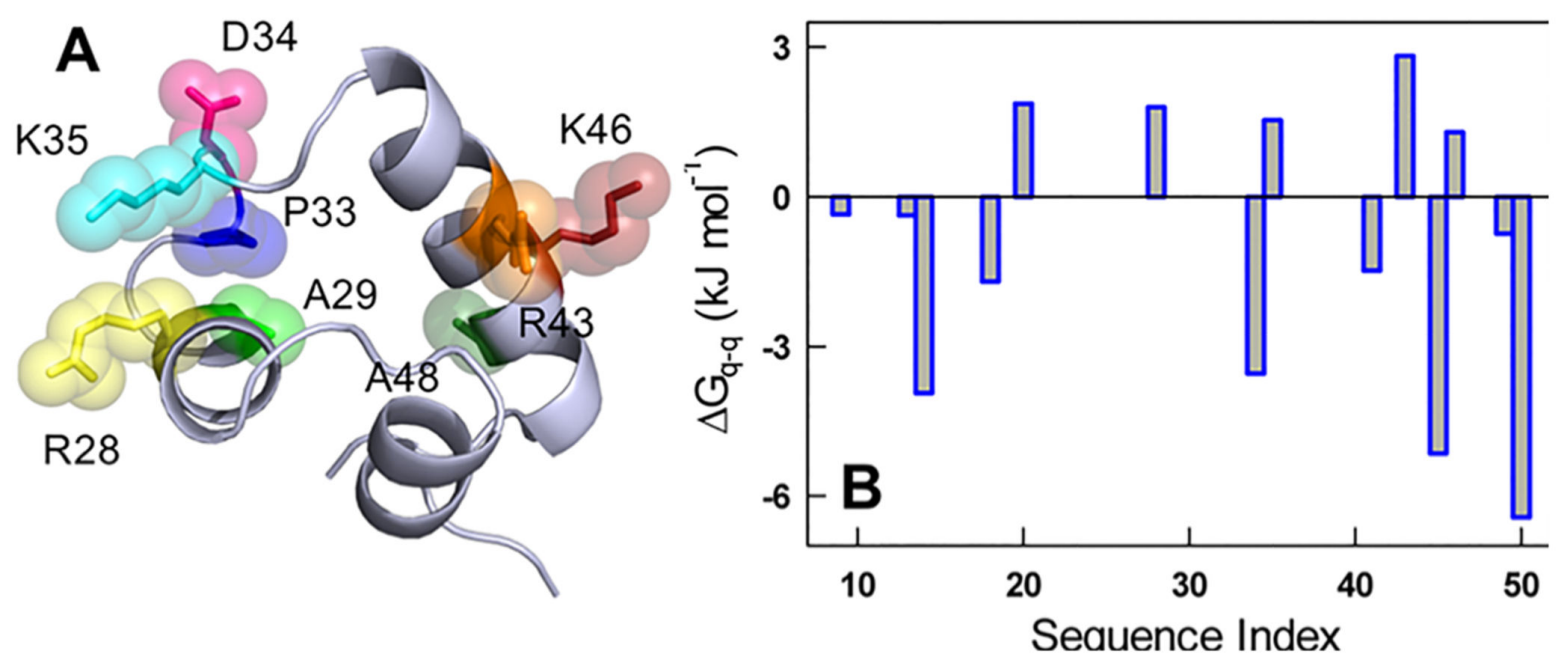
(A) Structure of the folded conformation of the CytR DNA-binding domain highlighting residues that are mutated in the current study. (B) Residue-wise charge–charge interaction energy as calculated from the Tanford–Kirkwood algorithm.^10,11^

**Figure 2:**
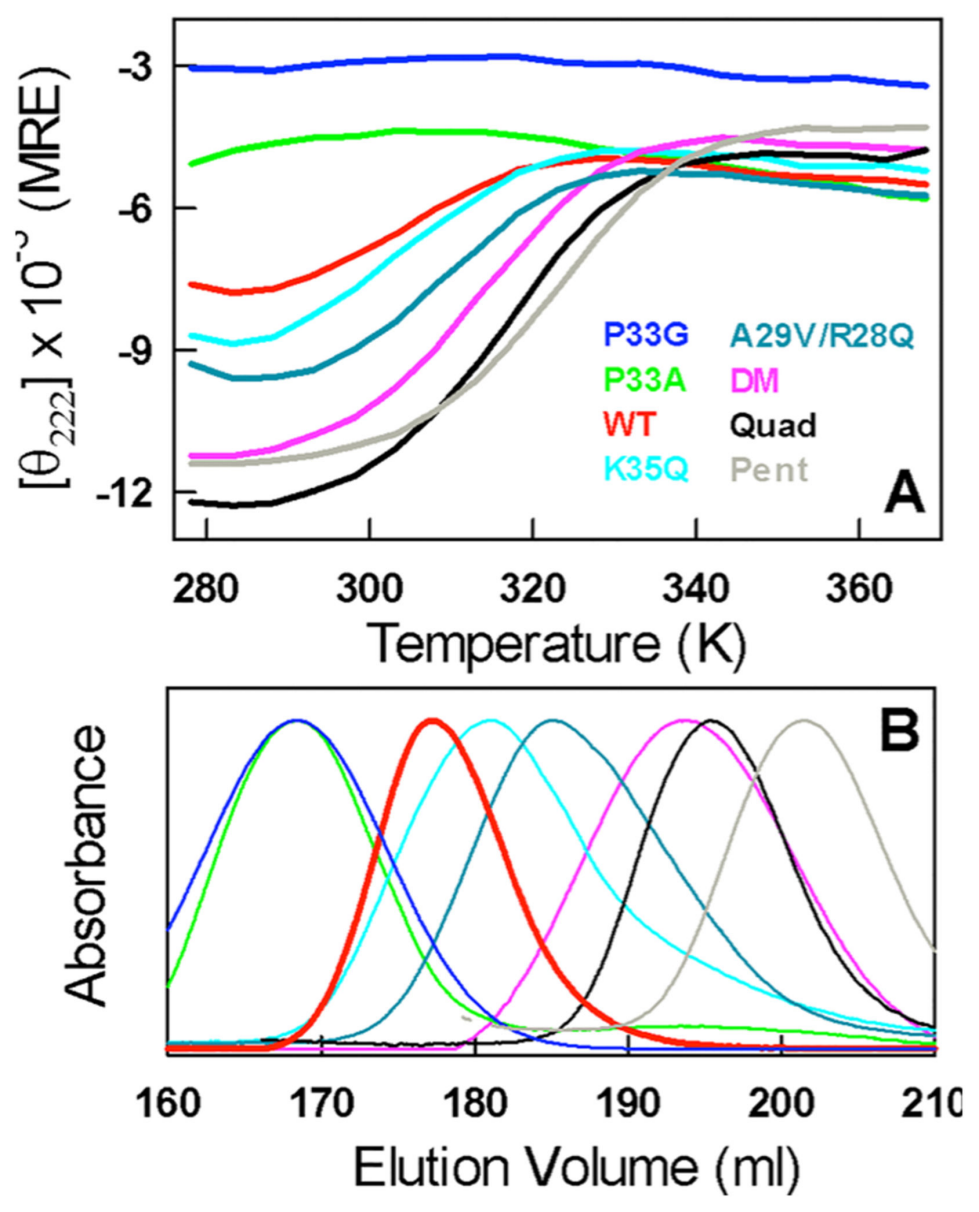
(A) Far-UV CD thermal unfolding curves of CytR and its mutants in 20 mM sodium phosphate buffer, pH 7.0, in mean residue ellipticity units of deg. cm^2^ dmol^−1^, as described in ref 20. (B) Size-exclusion chromatograms of the mutants in panel A at 298 K, 150 mM ionic strength ammonium acetate buffer at pH 8.0.^20^ A high ionic strength is required to eliminate nonspecific interactions of proteins with the column matrix. The reported dimensions are thus lower estimates as CytR reduces its dimensions with salt.^19^

**Figure 3:**
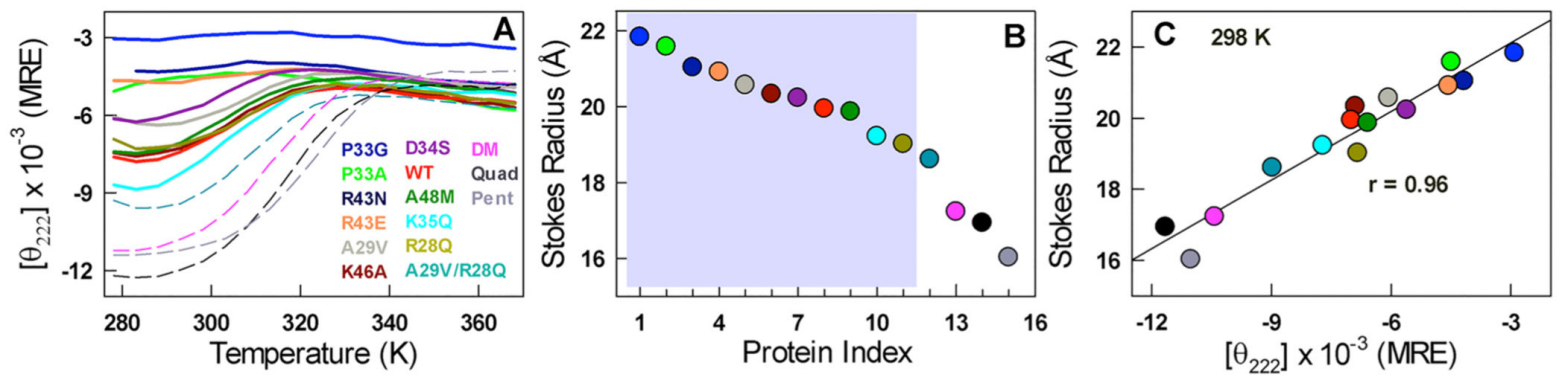
(A) Far-UV CD thermal unfolding curves of WT CytR (red), single-point mutants (continuous curves), and multiple-point mutants (dashed curves). (B) Estimated apparent Stokes radius following the ordering and color code in panel A. The shaded region represents the disordered regime. (C) Correlation between apparent molecular dimensions (ordinate) and the secondary structure (abscissa).

**Figure 4:**
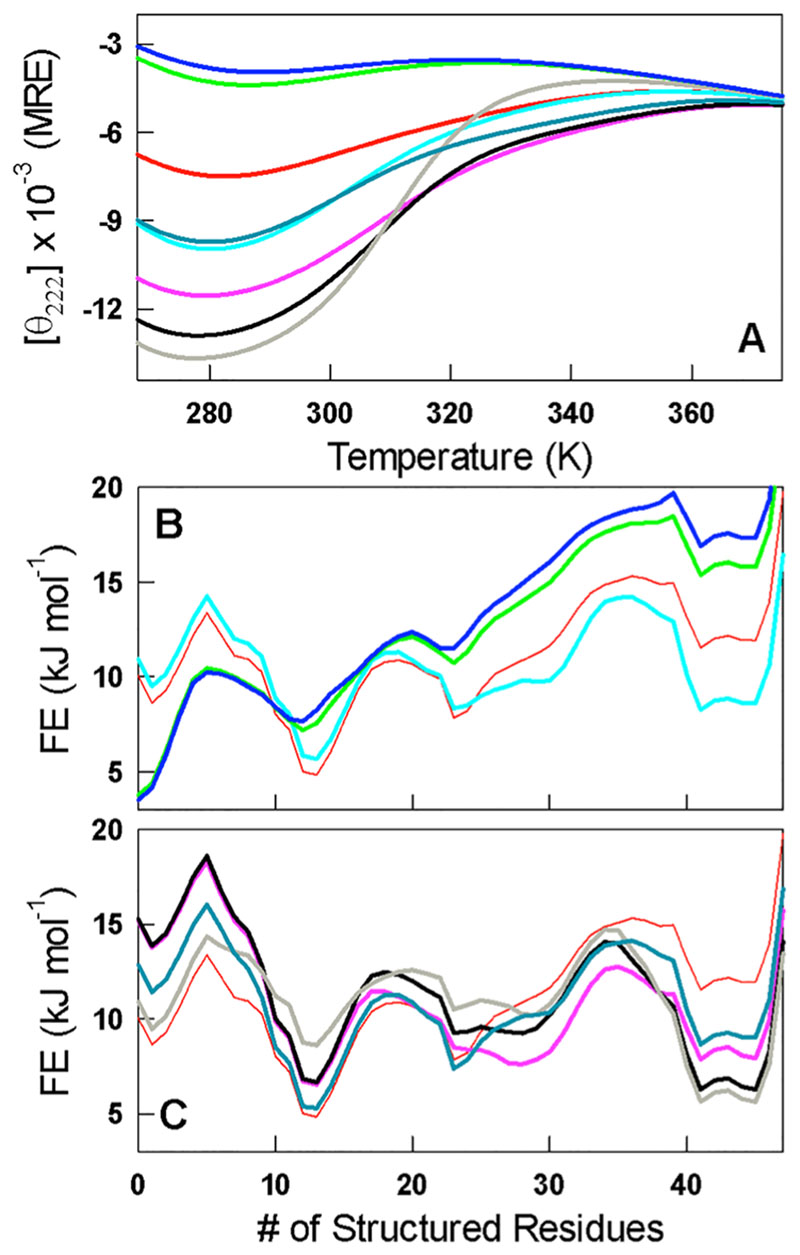
(A) Thermal unfolding curves predicted by the WSME model for the mutants of CytR following calibration of model parameters with the WT unfolding curve as described before.^21,29^ The color coding is the same as in Figure 3A. (B and C) Predicted one-dimensional free-energy profiles of WT CytR (thin red), single-point mutants (panel B), and multiple-point mutants (panel C).

## Abbreviations

CytR: cytidine repressor
WT: wild-type
CD: circular dichroism
MRE: mean residue ellipticity
*R*_s_: Stokes radius
IDP: intrinsically disordered protein
WSME: Wako–Saitô–Muñoz–Eaton
