# Supplementary material for: Controlling Structure and Dimensions of a Disordered Protein via Mutations
Source: Biochemistry. Author manuscript; Available in PMC 2020 Aug 14. (PMC7115935; doi:10.1021/acs.biochem.9b00678)
Supplement: Supporting Information [file EMS88735-supplement-Supporting_Information.pdf]

Supporting Information

Controlling Structure and Dimensions of a Disordered Protein *via* Mutations

Sneha Munshi, Divya Rajendran, Samyuktha Ramesh, Sandhyaa Subramanian,

*Kabita Bhattacharjee, Meagha Ramana Kumar & Athi N. Naganathan**

Department of Biotechnology, Bhupat & Jyoti Mehta School of Biosciences, Indian Institute of Technology Madras, Chennai 600036, India.

e-mail: athi@iitm.ac.in

Table S1 Parameters* are fixed from WSME model fits to the experimental far-UV CD melt of CytR WT employing LacR unfolding curve as a reference as described before.[1, 2] 298 K is used as the reference temperature for both the linear baselines. Mutations are introduced using PyMol and by merely choosing the best rotamer. The unfolding curves of all mutants are predicted by fixing the model parameters to that of the WT except for the ones noted below.

	ΔS_{conf} (J mol^{-1} K^{-1}) (all residues except position 33)	ΔS_{conf} (J mol^{-1} K^{-1}) (for residue 33)	ξ (J mol^{-1})	ΔC_p^{cont} (J mol^{-1} K^{-1})	*a*	*b*	*c*	*d*
**WT CytR and all mutants except P33A and P33G****	-34.0	0	-213.0	-2.1	-15.3	0.03	-1.65	-0.04
**P33A*****	-34.0	-34.0	-213.0	-2.1	-15.3	0.03	-1.65	-0.04
**P33G*****	-34.0	-40.1	-213.0	-2.1	-15.3	0.03	-1.65	-0.04

* **ΔS_{conf}** is the entropic penalty for fixing a residue in native conformation. **ξ** is the van der Waals interaction energy per native contact (derived from a 5 Å heavy-atom cut-off radius and including nearest neighbor interactions). ΔC_p^{cont} is the heat-capacity change per native contact that implicitly accounts for solvation effects. The parameters *a/c* and *b/d* are the intercepts and slopes of the folded (*F*) and unfolded (*U*) linear baselines, respectively, in mean residue ellipticity units (MRE; scaled down by a factor of 1000). The effective dielectric constant determining charge-charge interaction energy magnitudes is fixed to 29 from detailed thermodynamic analysis of the homologous protein unfolding[3] and stability modulations induced by mutations involving charged residues.[4]

** Since proline has restricted flexibility, we modeled it as requiring zero entropic penalty for fixing in native conformation as before.[2]

*** Mutating P33 to alanine is effectively represented as requiring a larger entropic penalty. P33G mutation is modeled as requiring a higher entropic penalty based on reported relative conformational entropies[5] and the excess conformational entropies of disordered residues (6.1 J mol^{-1} K^{-1} per residue).[6]

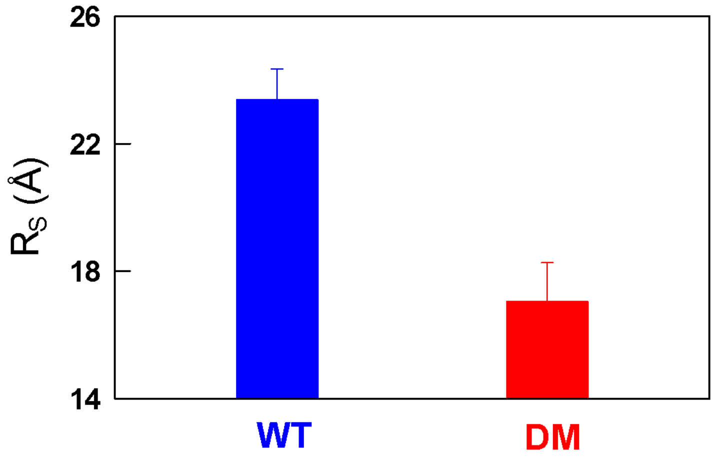

Figure S1 Dimensions of CytR WT and the double mutant (DM, A29V/A48M) from DLS measurements at 293 K.[7, 8]

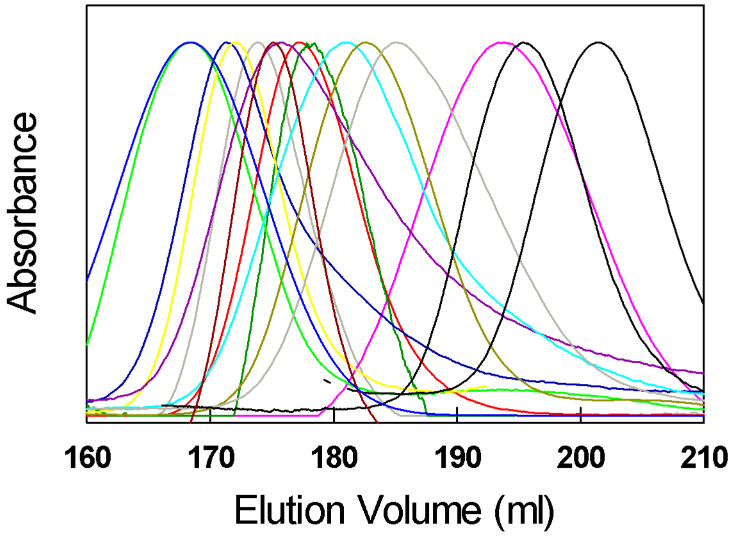

Figure S2 Size-exclusion chromatography (HiLoad 16/600 Superdex 75pg column) elution profiles for the CytR variants at 298 K (150 mM ionic strength, pH 8.0). The colors represent P33G (blue), P33A (green), R43N (dark blue), R43E (orange), A29V (gray), K46A (dark red), D34S (dark pink), WT CytR (red), A48M (dark green), K35Q (cyan), R28Q (dark yellow), A29V/R28Q (dark cyan), DM (magenta), Quad (black), and Pent (dark gray).

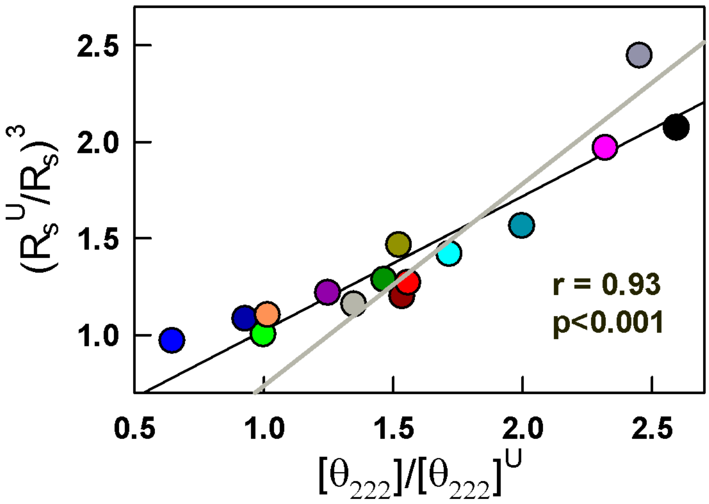

Figure S3 Ratio of relative apparent protein volumes (ordinate) and relative structure (abscissa) for the mutants of CytR at 298 K. Color coding is the same as in Figure 3A with the linear correlation shown in black. The mean-residue ellipticity and Stokes radius of the P33A mutant is used as a reference. The Uversky-Fink relation[9] is in gray. The circles represent P33G (blue), P33A (green), R43N (dark blue), R43E (orange), A29V (gray), K46A (dark red), D34S (dark pink), WT CytR (red), A48M (dark green), K35Q (cyan), R28Q (dark yellow), A29V/R28Q (dark cyan), DM (magenta), Quad (black), and Pent (dark gray).

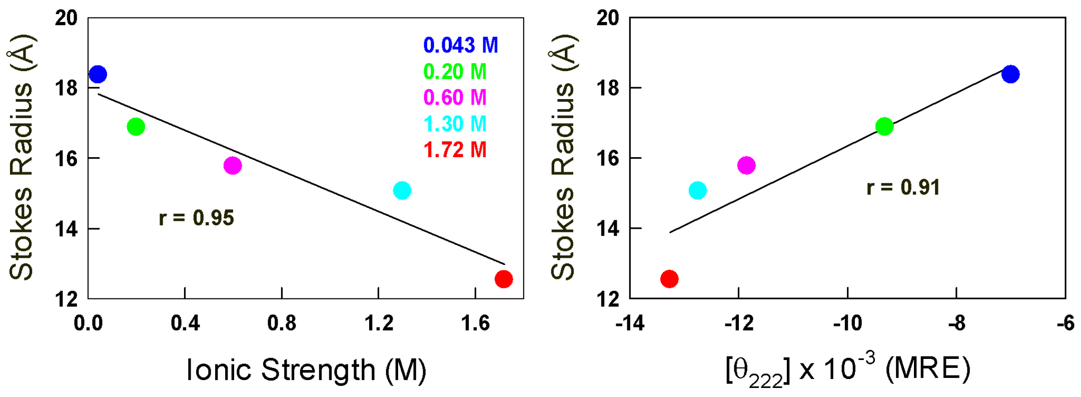

Figure S4 Left panel: Correlation between ionic strength (that induces structure) and Stokes radius for CytR WT. The dimensions at 0.043 M and 1.72 M are extracted from analytical ultracentrifugation[10] (AUC) while the rest are from size-exclusion chromatography. Right panel: Correlation between the Stokes radius and secondary structure following the same color code as in the left panel.

Supporting References

[1] Naganathan, A. N., and Orozco, M. (2013) The conformational landscape of an intrinsically disordered DNA-binding domain of a transcription regulator, *J. Phys. Chem. B 117*, 13842-13850.

[2] Munshi, S., Rajendran, D., and Naganathan, A. N. (2018) Entropic Control of an Excited Folded-Like Conformation in a Disordered Protein Ensemble, *J. Mol. Biol. 430*, 2688-2694.

[3] Naganathan, A. N. (2012) Predictions from an Ising-like Statistical Mechanical Model on the Dynamic and Thermodynamic Effects of Protein Surface Electrostatics, *J. Chem. Theory Comput. 8*, 4646-4656.

[4] Naganathan, A. N. (2013) A Rapid, Ensemble and Free Energy Based Method for Engineering Protein Stabilities, *J. Phys. Chem. B 117*, 4956-4964.

[5] Daquino, J. A., Gomez, J., Hilser, V. J., Lee, K. H., Amzel, L. M., and Freire, E. (1996) The magnitude of the backbone conformational entropy change in protein folding, *Proteins 25*, 143-156.

[6] Rajasekaran, N., Gopi, S., Narayan, A., and Naganathan, A. N. (2016) Quantifying Protein Disorder through Measures of Excess Conformational Entropy, *J. Phys. Chem. B 120*, 4341-4350.

[7] Munshi, S., Gopi, S., Subramanian, S., Campos, L. A., and Naganathan, A. N. (2018) Protein plasticity driven by disorder and collapse governs the heterogeneous binding of CytR to DNA, *Nucleic Acids Res. 46*, 4044-4053.

[8] Munshi, S., Subramanian, S., Ramesh, S., Golla, H., Kalivarathan, D., Kulkarni, M., Campos, L. A., Sekhar, A., and Naganathan, A. N. (2019) Engineering Order and Cooperativity in a Disordered Protein, *Biochemistry 58*, 2389-2397.

[9] Uversky, V. N., and Fink, A. L. (2002) The chicken-egg scenario of protein folding revisited, *FEBS Lett. 515*, 79-83.

[10] Munshi, S., Gopi, S., Asampille, G., Subramanian, S., Campos, L. A., Atreya, H. S., and Naganathan, A. N. (2018) Tunable order-disorder continuum in protein-DNA interactions, *Nucleic Acids Res. 46*, 8700-8709.
